# Supplementary material for: Content-rich biological network constructed by mining PubMed abstracts
Source: BMC Bioinformatics. 2004 Oct 8;5:147. doi: 10.1186/1471-2105-5-147 (PMC528731; doi:10.1186/1471-2105-5-147)
Supplement: Additional File 2 — The original results of the above study (non-essential files are deleted to keep the file size under the limit set by BMC bioinformatics). [file 1471-2105-5-147-S2.bz2 › chilibotAdditionalFile2/dip30/246ID8962075E809/html/RECO_RECF.html]

 


 **RECO** and **RECF** 
  
Found 63 abstracts in PubMed, retrieved 30.  
 

 What does Google say? 
 PDF only 
| .edu only 

---

**Interactive relationship** (e.g. stimulation, inhibition, etc)

**Neutral relationship**- **RecF**  protein competes with  **RecO**  protein for RecR protein association and its addition destabilizes RecAOR filaments.  Ref: 11743007 EMBO J, 2001
- Finally, we found that interactions of  **RecF**  with  **RecO**  are lost in the presence of ATP.  Ref: 8962075 Proc Natl Acad Sci U S A, 1996
- These data suggested that  **RecO**  mediates the interactions of  **RecF**  protein with RecR and with Ssb proteins.  Ref: 8962075 Proc Natl Acad Sci U S A, 1996
- The  **recO**  gene is required for the  **RecF**  pathway of recombination and the repair of DNA daughter strand gaps in Escherichia coli.  Ref: 8538645 Mutat Res, 1996
- Since  **recF**  is epistatic with  **recO**  and recR recOR for UV resistance, recOR mutations were also tested with priA2 kan.  Ref: 8820655 Mol Microbiol, 1996
- iii  **RecF**  interacts with Ssb protein in the presence of  **RecO** .  Ref: 8962075 Proc Natl Acad Sci U S A, 1996
- These data suggest that one role of  **RecF**  is to stabilize the interaction of RecR with  **RecO**  in the presence of Ssb protein.  Ref: 8962075 Proc Natl Acad Sci U S A, 1996
- No synergistic interactions were detected between helD and the  **recF** ,  **recO**  and regN genes when conjugational recombination was analyzed.  Ref: 8647383 Genetics, 1995
- We observed synergistic interactions between uvrD ant the  **recF** , recN,  **recO**  and recG genes in both conjugational recombination and the repair of methylmethane sulfonate MMS induced DNA damage.  Ref: 8647383 Genetics, 1995
- We did detect synergistic interactions between helD and  **recF**   **recO**  in recombinational repair.  Ref: 8647383 Genetics, 1995
- The  **recF** ,  **recO** , and recR genes were originally identified as those affecting the  **RecF**  pathway of recombination in Escherichia coli cells.  Ref: 9537373 J Bacteriol, 1998
- We therefore assigned  **recO**  to epistatic group alpha and predict that the  **RecO**  protein acts at the same stage of recombination as the  **RecF** , RecL and RecR proteins, in a RecFLOR complex.  Ref: 10323239 Mol Gen Genet, 1999
- Incubation of  **RecF** ,  **RecO** , RecR, and Ssb proteins resulted in the formation of  **RecF**   **RecO**  Ssb complexes ; i.e., RecR was excluded.  Ref: 8962075 Proc Natl Acad Sci U S A, 1996
- Interactions of  **RecF**  protein with  **RecO** , RecR, and single stranded DNA binding proteins reveal roles for the  **RecF**   **RecO**  RecR complex in DNA repair and recombination.  Ref: 8962075 Proc Natl Acad Sci U S A, 1996
- Preincubation of  **RecF** ,  **RecO** , and RecR proteins prior to addition of Ssb protein resulted in the formation of complexes consisting of  **RecF** ,  **RecO** , RecR, and Ssb proteins.  Ref: 8962075 Proc Natl Acad Sci U S A, 1996
- On the other hand, the presence of recA2020, a suppressor mutation for  **recF** ,  **recO** , and recR mutations, restored normal UVM in  **recF** ,  **recO** , and recR mutants.  Ref: 9537373 J Bacteriol, 1998

**Non-interactive relationship** (e.g. studied together, co-existance, homology, etc.)

- This action of RecA does not involve the induction of the SOS response and does not require any of the recombination proteins essential for the presynaptic step of homologous recombination, RecBCD,  **RecF**  or  **RecO** .  Ref: 11069680 Mol Microbiol, 2000
- These results indicate an involvement of the  **recF** ,  **recO** , and recR genes and their products in UVM, possibly by affecting the third role of RecA in UVM.  Ref: 9537373 J Bacteriol, 1998
- In Escherichia coli, the recovery of replication following UV induced DNA damage is dependent upon several proteins in the  **recF**  pathway, including  **RecF** ,  **RecO** , and RecR.  Ref: 10589843 Mol Gen Genet, 1999
- Gap repair genes  **recF** ,  **recO** , and recR were not required.  Ref: 10224240 Genetics, 1999
- The protective effect of plasmid pGam18 against radiation was completely abolished by mutations in the most  **RecF**  pathway genes  **recF** , recJ, recR,  **recO** , recQ, recN, and ruvB.  Ref: 10368783 Genetika, 1999
- The defects in restart attributed to mutations in  **recF** ,  **recO**  or recR were suppressed in a recA730 lexA Def strain expressing constitutively activated RecA RecA\*.  Ref: 11929519 Mol Microbiol, 2002
- Several lines of evidence suggest that the  **recF** ,  **recO** , and recR genes function at the same step of recombination and postreplication repair.  Ref: 9537373 J Bacteriol, 1998
- The results of these studies suggest that recombinational repair genes, particularly  **recF** , sbcB and  **recO**  have different roles in the induced excision of Tn10 than in recombinational mating.  Ref: 10102036 Mutat Res, 1999
- We discuss these results to explain how the  **RecF**   **RecO**  RecR complex functions as an anti Ssb factor.  Ref: 8962075 Proc Natl Acad Sci U S A, 1996
- The  **recO**  mutation did not affect the sensitivity of  **recF** , recR or recL cells to DNA damaging agents, increased the sensitivity of recU and addAB cells and abolished the DNA repair capacity of recH cells.  Ref: 10323239 Mol Gen Genet, 1999
- Roles for lambda Orf and Escherichia coli  **RecO** , RecR and  **RecF**  in lambda recombination.  Ref: 9335578 Genetics, 1997
- Expression of the Escherichia coli mutA mutator phenotype requires recA, recB, recC, ruvA, and ruvC gene, but not recD,  **recF** ,  **recO** , or recR genes.  Ref: 10671469 J Bacteriol, 2000
- In this study, we find that other genes belonging to the RecBC and  **RecF**  sexual recombination pathways also participate in this process since mutations in recB, sbcB or  **recO**  diminish, though to different degrees, the frequency of Tn10 precise excision induced by MMC treatment or by uvrD mutants.  Ref: 10102036 Mutat Res, 1999
- Resistance of E. coli strains to cisplatin as determined by plating experiments decreased in the following order parent PQ30 strain, polB >  **recO** , recR,  **recF**  > recB.  Ref: 12531023 DNA Repair Amst , 2002
- In this work, we report that null mutations in  **recF** ,  **recO** , or recR greatly reduce UV radiation mutagenesis UVM in an assay for reversion from a Trp trpE65 to a Trp phenotypes.  Ref: 9537373 J Bacteriol, 1998
- The Escherichia coli  **RecF** ,  **RecO**  and RecR pro teins have previously been implicated in bacterial recombinational DNA repair at DNA gaps.  Ref: 11743007 EMBO J, 2001
- ii In a recBC sbcA background, in which RecE mediated recombination is active, recA, recJ, recQ, recE, recT,  **recF** ,  **recO** , and recR mutations led to their accumulation.  Ref: 12718760 BMC Mol Biol< MedlineTA>BMC Mol Biol, 2001
- Genetic evidence suggests that the  **RecF** ,  **RecO** , and RecR RecFOR proteins participate in a common step of DNA recombination and repair, yet the biochemical event requiring collaboration of all three proteins is unknown.  Ref: 12769856 Mol Cell, 2003
- Introduction of the defective lexA51 mutation lexA51 Def and or UmuD into  **recF** ,  **recO** , and recR mutants failed to restore normal UVM in the mutants.  Ref: 9537373 J Bacteriol, 1998
- Involvement of  **recF** ,  **recO** , and recR genes in UV radiation mutagenesis of Escherichia coli.  Ref: 9537373 J Bacteriol, 1998
- dnaC suppressors of priA overcome this inviability, especially when  **RecF** ,  **RecO**  or RecR is inactivated, indicating that RdgC avoids or counters a toxic effect of these proteins.  Ref: 12554673 EMBO J, 2003
- Although the MRF alpha 2 components are apparently not encoded by currently known homologous recombination genes such as recA,  **recF** ,  **recO** , and recR, they may fulfill an important function in assembling replisomes on arrested replication forks and products of homologous strand exchange.  Ref: 11459960 Proc Natl Acad Sci U S A, 2001
- a dsRNA endonuclease involved in rRNA and mRNA processing and decay, Era an essential G protein of unknown functions and  **RecO**  involved in the  **RecF**  homologous recombination pathway.  Ref: 9150881 Biochimie, 1996
- The  **recO**  mutation did not affect intermolecular recombination in  **recF** , recL, recH or recU cells, but reduced by about 9 fold the incidence of intermolecular recombination in addAB cells.  Ref: 10323239 Mol Gen Genet, 1999
- These observations are incorporated into an expanded model for the functions of  **RecF** ,  **RecO** , and RecR proteins in the early stages of postreplication DNA repair.  Ref: 9363943 Cell, 1997
- The  **recO**  mutation did not affect intramolecular recombination in the addAB, recU,  **recF**  or recL cells, but reduced it by about 75 fold in recH cells.  Ref: 10323239 Mol Gen Genet, 1999
- Models for the roles of Orf and  **RecO** , RecR and  **RecF**  in recombination are presented.  Ref: 9335578 Genetics, 1997
- ii this pathway s requires functional recA,  **recF** ,  **recO** , and recR genes, suggesting the mechanism to be daughter strand gap repair.  Ref: 11073901 J Bacteriol, 2000
- Expression of a gene s from the nin region of phage lambda partially complemented both the viability and recombination defects of the  **recF** ,  **recO** , and recR mutants and the recombination defect of ruvC but not of ruvAB or recQ mutants.  Ref: 10735883 J Bacteriol, 2000
- A  **recO**  deficient strain did not exhibit dramatic reduction in efficiency of IRR, implying that  **RecF**  and RecR proteins maintain function during this mechanism in the absence of functional  **RecO** .  Ref: 8921869 Gene, 1996
- Finally, examples are shown for in vitro reactions involving DNA, RecA,  **RecO** ,  **RecF** , RecR, and SSB that lead to a further understanding of recombinational repair.  Ref: 11098470 Mol Biotechnol, 2000
- The products of the  **recF** ,  **recO** , and recR genes are thought to interact and assist RecA in the utilization of single stranded DNA precomplexed with single stranded DNA binding protein Ssb during synapsis.  Ref: 8962075 Proc Natl Acad Sci U S A, 1996
- To better understand the role of these two proteins in homologous recombination in the  **RecF**  pathway recBCsbcB C background, we investigated the interactions between helD, uvrD and the following  **RecF**  pathway genes  **recF** ,  **recO** , recN and ruvAB.  Ref: 8647383 Genetics, 1995
- We compared the recombination proficiencies of recB1080,  **recO** , recR, and  **recF**  single mutants and recB1080  **recO** , recB1080 recR, and recB1080  **recF**  double mutants.  Ref: 12618388 Genetics, 2003
- Similarly,  **recF** ,  **recO**  or recR mutations suppress the lethality of the Escherichia coli rep uvrD double mutant.  Ref: 12065426 EMBO J, 2002
- An increase of intracellular  **RecO** , which may be due to the increased  **recO**  gene copies or to the induction of  **RecO**  synthesis, increased the UV resistance of recA,  **recF** , and ssb cells, but did not increase the UV resistance of uvrB, uvrB  **recF** , uvrB recA and uvrB ssb cells.  Ref: 8538645 Mutat Res, 1996
- In a wild type, lexA background, mutations in  **recF** ,  **recO**  or recR block both pathways.  Ref: 11929519 Mol Microbiol, 2002
- To elucidate the role of several key proteins involved in this process, we have analysed the time dependence of replication restart in strains carrying a combination of mutations in lexA, recA, polB pol II, umuDC pol V, priA, dnaC,  **recF** ,  **recO**  or recR.  Ref: 11929519 Mol Microbiol, 2002
- previously isolated mutations in B.subtilis  **recF** , recL,  **recO**  and recR, which belong to the same complementation group, all suppress the lethality of a pcrA mutation.  Ref: 12065426 EMBO J, 2002
- Bacteriophage lambda lacking its Red recombination functions requires either its own gene product, Orf, or the product of Escherichia coli s  **recO** , recR and  **recF**  genes RecORF for efficient recombination in recBC sbcB sbcC mutant cells the  **RecF**  pathway.  Ref: 9335578 Genetics, 1997
- This function is countered by the  **RecF**   **RecO**  competition for association with the RecR protein.  Ref: 11743007 EMBO J, 2001
- Using immunoprecipitation, size exclusion chromatography, and Ssb protein affinity chromatography in the absence of any nucleotide cofactors, we have obtained the following results i  **RecF**  interacts with  **RecO** , ii  **RecF**  interacts with RecR in the presence of  **RecO**  to form a complex consisting of  **RecF** ,  **RecO** , and RecR  **RecF**   **RecO**  RecR.  Ref: 8962075 Proc Natl Acad Sci U S A, 1996
- In this genetic background, mutations in  **recF** ,  **recO** , and recR had large effects on both cell viability and on recombination.  Ref: 10735883 J Bacteriol, 2000
